# Gut microbiota and pro/prebiotics in Alzheimer’s disease

**DOI:** 10.18632/aging.102930

**Published:** 2020-03-19

**Authors:** Ryszard Pluta, Marzena Ułamek-Kozioł, Sławomir Januszewski, Stanisław J. Czuczwar

**Affiliations:** 1 Laboratory of Ischemic and Neurodegenerative Brain Research, Mossakowski Medical Research Centre, Polish Academy of Sciences, Warsaw, Poland; 2 Department of Pathophysiology, Medical University of Lublin, Lublin, Poland

**Keywords:** Alzheimer’s disease, gut-brain-microbiota axis, gut microbiota, probiotics, prebiotics

## Abstract

Alzheimer’s disease is characterized by the accumulation of amyloid and dysfunctional tau protein in the brain along with the final development of dementia. Accumulation of amyloid in the brain was observed 10-20 years before the onset of clinical symptoms by diagnostic methods based on image analysis. This is a serious public health problem, incidence and prevalence being expected to reach epidemic proportions over the next few decades if the disease cannot be prevented or slowed down. Recently, in addition to the strongly developing ischemic etiology of Alzheimer’s disease, it is suggested that the gut microbiota may also participate in the development of this disease. The brain and gut are thought to form a network called the “gut-brain-microbiota axis”, and it is strongly supported idea that the intestinal microflora can be involved in Alzheimer’s disease. Lately, many new studies have been conducted that draw attention to the relationship between Alzheimer’s disease and gut microbiota. This review presents a possible relationship between Alzheimer’s disease and a microbiome. It is a promising idea for prevention or therapeutic intervention. Modulation of the gut microbiota through a personalized diet or beneficial microflora intervention like pro/prebiotics, changing microbiological partners and their products, including amyloid protein, can become a new treatment for Alzheimer’s disease.

## INTRODUCTION

The cause of Alzheimer’s disease is currently unknown, but it has been shown that the onset of the disease occurs 10-20 years before the onset of clinical symptoms and includes various factors that are ultimately not defined [1]. Factors that may be involved in the development of the disease are thought to include lifestyle habits such as diet, exercise, education history, cognition and aging, immunosenescence, chronic infections, chronic inflammation, latent infections, sleep problems and other [1, 2]. A randomized clinical trial was reported in which sleep in healthy middle-aged men reduced cerebrospinal fluid amyloid levels and lack of sleep counteracted this decrease [2]. In a study assessing the relationship between the Mediterranean diet and dementia development, it was noted that the traditional Mediterranean diet, which consists of a large number of vegetables, fruits and cereals, reduced the risk of developing Alzheimer’s disease and dementia [3]. In addition, Ozawa et al., [4] observed over 1,000 patients with Alzheimer’s disease for 17 years, and found that the incidence of Alzheimer’s disease decreased significantly with the increase in the consumption of milk and dairy products. Lifestyle therefore plays an important role in preventing Alzheimer’s disease, and lifestyle dysregulation may not only lead to Alzheimer’s disease, but also to various other health problems such as dysregulation of the gut microbiota. The composition of symbiotic microorganisms has changed dramatically throughout human history with the development of agriculture, industrialization and globalization. It is postulated that each of these lifestyle changes resulted in a gradual disappearance of microbial diversity and an increase in their virulence, thus causing the formation of a risk path for Alzheimer’s disease pathogenesis. Changes in the microbial composition throughout history suggest an escalation of the risk of Alzheimer’s disease. Recent advances in research on the etiology of Alzheimer’s disease suggest that microbiota (oral, nasal, intestinal) dysbiosis during life can lead to a systemic inflammatory response and affect microglia immune response in the brain. More and more experimental and clinical data confirm the key role of intestinal dysbiosis and interaction of the intestinal microflora with the host in the development of neurodegeneration [5]. What is more, over time, the pathological permeability of the intestinal mucosa and blood-brain barrier begins to increase and a *vicious circle* is formed that irreversibly destroys neurons. It is likely that the convergence of the inflammatory response from the gut along with aging and poor diet in the elderly contributes to the pathogenesis of Alzheimer’s disease. Modifying the composition of the intestinal microflora with food-based therapy or pro/prebiotic supplementation can create new preventive and therapeutic options for Alzheimer’s disease. The future of pro/prebiotic in Alzheimer’s disease depends on the progress of research on the role of intestinal microflora in the development of Alzheimer’s disease. We must first understand how and when intestinal bacteria promote Alzheimer’s disease. This review aims to highlight the role of intestinal microflora in the onset and progression of Alzheimer’s disease.

## Gut microbiota versus brain

The relationship between the gut microflora and the brain is that the intestine and the brain can interact with each other *via* the nervous system or chemicals that cross the blood-brain barrier. For example, the vagus nerve connects intestinal nerve cells with neurons in the brain [6]. The intestinal flora produce, i.e. monoamines, methionine, glutamate and homocysteine, which *via* the lymphatic and circulatory system reach the central neurons and can affect their activity, which may manifest as behavioral changes [7, 8]. On top of it, intestinal bacteria are sensitive to information sent by the brain *via* neurotransmitters [8, 9]. The vagus nerve with its own nuclei in the brainstem serves as a connection between the intestines and the spinal cord through the incoming and outgoing fibers [10]. In this situation, the brainstem nuclei can monitor different bowel functions and spread signals to other areas of the brain such as the thalamus and cerebral cortex [11]. To sum up, the “gut-brain-microbiota axis” is a bottom-up concept, in contrast to the top-down term “brain-gut-microbiota axis”’, no matter what it is called, its meaning refers to two-way communication between the intestine and the brain [11]. To top it all off, the intestinal nervous system can exchange information with the brain *via* intestinal bacteria [12]. Exchange of information and substances between the intestine and the brain can also occur *via* the peripheral circulatory system [13]. The intestinal mucosa and blood-brain barrier allow the passage of cytokines and hormones that can affect both intestinal and brain tissue [14]. In germ-free mice, intestinal bacteria have been documented to affect the maturation of the nervous, endocrine and immune systems [11]. The brain-gut-microbiota axis is considered as a multifunctional network in which the central, peripheral, hormonal and immune systems participate in two-way communication [15]. The intestinal microflora is able to synthesize and release neuromodulators and neurotransmitters such as glutamate, short chain fatty acids, biogenic amines, serotonin, dopamine and histamine and other amino acid metabolites such as homocysteine, GABA and tryptophan [8, 16]. All these molecules act in the brain tissue and control the activity of neurons. Studies have indeed confirmed that microflora changes are responsible for behavioral abnormalities, but have not revealed any direct cause-effect [17]. Another possibility is that the intestinal microflora produces neurotoxic substances such as D-lactic acid, homocysteine, pro-inflammatory cytokines and ammonia, which are subsequently released into the brain [8, 18, 19]. Thus, the intestinal microflora can affect the brain-gut-microbiota axis *via* immune, neuroendocrine and direct nerve mechanisms [13]. The above changes can cause anxiety, memory impairment and other cognitive disorders [17, 18, 20, 21]. According to the latest research, changes in the intestinal microflora are associated with various neurodegenerative diseases [22], and among neurodegenerative diseases there is evidence of possible involvement of intestinal dysbiosis in the development of Alzheimer’s disease [23].

## Gut microbiota versus Alzheimer’s disease

Suggestions that the intestinal microflora may be invos disease lved in the neuropathology of Alzheimer’s disease are mainly from experimental research. That is why germ-free animals are used to study the effect of intestinal microflora on brain pathology. A significant reduction in amyloid accumulation and its neurotoxicity has been observed in thse rodents and these negative eeffects occur again when the animals are exposed to the intestinal microflora of control mice [24].

A study comparing the microbiota of 25 Alzheimer’s disease cases with 25 controls showed a reduced microbial diversity in Alzheimer’s disease patients [25]. In addition, a decrease in the number of *Firmicutes* and an increase in the percentage of *Bacteroidetes* were observed [25]. Another study comparing the microbiome of non-demented patients with dementia patients found that *Bacteroides* were reduced in patients with dementia compared with non-dementia patients [26]. In addition, cultivable butyrate-producing bacteria involved in cognitive function have been isolated from the microbiota of Japanese Alzheimer’s disease patients [27]. The next investigation provided evidence that in the study of Alzheimer’s disease and mild cognitive impairment patients from China and healthy people, microbiological diversity of feces was reduced in patients with Alzheimer’s disease compared to patients with mild cognitive impairment and healthy subjects [28]. In addition, there was also a decrease in the number of *Firmicutes* and an increase in the number of *Proteobacteria* [28].

Many human studies have recently shown that bacterial or viral infection [29] can be one of the causes of Alzheimer’s disease. The effect of chronic *Helicobacter pylori* infection on Alzheimer’s disease has been demonstrated by the release of massive inflammatory mediators [30]. Plasma levels of β-amyloid peptides 1-40 and 1-42 increased in patients with Alzheimer’s disease infected with *Helicobacter pylori* or *Borrelia burgdorferi* and *Chlamydia pneumoniae* [31]. *Helicobacter pylori* filtrate *in vitro* induced hyperphosphorylation of tau protein of Alzheimer’s disease type by activation of glycogen-3β synthase kinase [32]. In addition, multi-bacterial infections were found in the brain tissue of Alzheimer’s disease patients [33]. In the hippocampus and temporal lobe lysates from the brains of patients with Alzheimer’s disease, a high level of bacterial lipopolysaccharide was found, which is an important internal factor contributing to inflammatory brain degeneration [34]. Serum analysis of people with cognitive impairment also showed elevated levels of pro-inflammatory cytokines, as well as higher pro-inflammatory (*Shigella/Escherichia*) and reduced anti-inflammatory intestinal microbiome (*Escherichia rectale*) [35]. *Herpes simplex virus type 1* has been documented as an important risk factor for the development of Alzheimer’s disease, and its research may reveal mechanisms and signposts in the search for the etiology of the disease [36]. Other viruses such as *cytomegalovirus* [37] and *varicella-zoster virus* [38, 39] have been also associated with Alzheimer’s disease, although their role as individual risk factors for the development of the disease is unclear [40]. The interaction between *cytomegalovirus* and *herpes simplex virus type 1* has been found to be significantly associated with the development of Alzheimer’s disease [40]. These data suggest that *cytomegalovirus* infection facilitates the development of Alzheimer’s disease associated with *herpes simplex virus type 1,* perhaps by affecting the immune system [40]. Some studies have indicated that the intestinal microflora can also affect proteins and receptors involved in synaptic plasticity, such as the NMDA receptor, brain-derived neurotrophic factor and serotonin receptors, in addition to serotonin alone [41]. Dysbiosis caused by a high fat diet can trigger neuroinflammation with the generation of pro-inflammatory cytokines and decline of immune regulating activity [42]. Under normal conditions, e.g. *Clostridium butyricum* has a neuroprotective effect by increasing the secretion of glucagon-like peptide-1 [43], and other intestinal bacteria produce, e.g. short chain fatty acids and antioxidants, which also protect the brain from pathogens [44].

## Gut microbiota amyloids versus amyloid generation in the brain

Some *Enterobacteria species* and/or fungi may produce amyloid peptides or a curly type amyloid fiber that is capable of forming seeding for aggregation of amyloid in the brain [45–48]. Microbial amyloids increase the nucleation of β-amyloid peptide aggregates [49] and trigger an inflammatory response [50]. On top of it, to nucleation of β-amyloid peptide aggregates, bacterial amyloid peptides also increase the aggregation of other misfolded proteins such as alpha-synuclein [49].

A reduction in amyloid accumulation was observed in APPPS1 transgenic mice in the absence of gut microbiota [24]. In addition, the microflora of the transgenic mouse model differs from the wild-type microflora, which causes amyloid accumulation in wild-type mice transplanted with the microbiome of the Alzheimer’s disease transgenic mouse model [24]. Short-chain fatty acids derived from the intestinal microflora strongly inhibit amyloid aggregation *in vitro* [51]. In addition, bacterial endotoxin may be involved in neuroinflammation associated with amyloid fibril formation in Alzheimer’s disease [52]. Although some bacteria, such as *Escherichia coli*, produce amyloid [53], but the association of this amyloid with neurodegenerative diseases, such as Alzheimer’s disease is not definitively explained [54]. Bacterial amyloid has been shown to activate signaling pathways that play a role in the pathogenesis of Alzheimer’s disease, and microflora is an important key player that expands neuroinflammation associated with the production and accumulation of amyloid [55]. In addition, bacterial gram-negative lipopolysaccharide promotes the accumulation of amyloid in the brain of mice, negatively affecting cognitive functions [56, 57].

However, it is not known how bacterial amyloids interact with other pathological processes in Alzheimer’s disease, such as tau protein dysfunction, neuroinflammation and cerebrovascular degeneration. Studies have shown that metabolites released from abundant bacteria in a healthy digestive tract maintain cognitive function, while metabolites released from pro-inflammatory bacterial species exacerbate Alzheimer’s disease by intensifying brain neuroinflammation. When the intestinal barrier has increased permeability, immunogenic bacterial amyloids enter the systemic circulation and aggravate neuroinflammation in the brain [34, 58]. These data show that bacterial amyloids may play an important role in exacerbating immunoreaction and nucleating amyloid aggregates in the brain. As a result of the interaction of microbial amyloids with microglia, bacterial amyloids appear to remove β-amyloid peptide aggregates by activated microglia [59]. The above suggestions have recently been supported by *in vivo* and *in vitro* studies [60, 61]. Thus microbial amyloids can control neuroinflammation and β-amyloid peptide levels by regulating reactive gliosis in the brain. Impaired bacterial flora can change the levels of bacterial amyloids and metabolites in the serum, and therefore may play a triggering role in the onset and exacerbation of neurodegeneration in Alzheimer’s disease.

## Gut microbiota versus behavioral changes

As already mentioned above, the human brain and gut form a network called the “brain-gut-microbiota axis”. Intestinal microflora has been shown to be a key element in this network. Mice were transplanted with different microflora, and their response to stress caused by adrenocorticotropic hormone and corticosterone was assessed [62]. In particular, compared to specific pathogen-free mice, adrenocorticotropic hormone and corticosterone levels have been reported to increase significantly in germ-free mice due to stress associated with restriction [62]. A similar effect is also seen in *Bifidobacterium infantis* transplanted mice, with a parallel effect on brain neurotransmitters. [63, 64]. Pregnant mice colonized with human commensal bacteria that induce intestinal Th17 cells have been shown to have offspring with increased anxiety behavior [65]. On the contrary, these anxiety behaviors were not observed if mothers were previously treated with interleukin-17a blocking antibody. In this way, it was demonstrated that the intestinal microflora of the mother mouse is involved in the behavior of the offspring. In addition, cognitive deterioration has been demonstrated in germ-free mice transplanted with microflora of Alzheimer’s disease patients [66].

## Gut microbiota versus amyloid and tau protein clearance

Intestinal microbiomes located in the colon and ileum produce biologically active short chain fatty acids that can pass the blood-brain barrier. A meta-analysis showed that intestinal metabolites can increase inflammation, aggregation of amyloid and tau protein in the brain [67]. Intestinal bacteria secrete over 100 metabolites, but their effect on the pathogenesis of Alzheimer’s disease has not been proven [68]. Valerian, isovaleric, isobutyric, butyric, propionic, acetic and formic acids have been studied and found to affect Alzheimer’s disease development by interfering with astrocyte and microglia activation, helping reduce inflammation, and aggregating tau protein and amyloid [69, 70]. A significant effect of the host microflora on microglia homeostasis was observed, which in germ-free mice caused global microglia defects with altered cell proportions and immature phenotype, leading to impaired innate immune responses [71]. In contrast, recolonization with complex microflora partly restored the characteristics of microglia. It has been established that short-chain fatty acids, bacterial products of bacterial fermentation, regulate microglia homeostasis. Therefore, mice deficient in the short-chain fatty acid FFAR2 receptor reflected microglia defects found in germ-free conditions. These data suggest that host bacteria naturally regulate microglia maturation and function, while microglia impairment can be somewhat remedied by complex microflora [71]. In summary, this study showed that the intestinal microflora can control microglia maturation, activation and function, and therefore, in cases of impaired intestinal microflora, microglia maturation and the possibility of tau protein and amyloid phagocytosis are drastically reduced. It should be added that short chain fatty acids serve as an alternative energy substrate for impaired energy metabolism in Alzheimer’s disease [72–74]. All this evidences indicate that intestinal microbes are necessary for microglia maturation and suppression of inflammation in the brain, which was also supported by epigenetic studies investigating the effects of short chain fatty acids on Alzheimer’s disease development [68].

Other secretory activity of microbes are neurotransmitters such as dopamine, acetylcholine, noradrenaline, gamma-aminobutyric acid, serotonin and histamine (by *Bacillus species*, *Bifidobacterium species*, *Enterococcus species*, and *Escherichia species*) [68]. *In vitro* study with photo induced cross-linking protocol of unmodified proteins found that propionic, butyric and valeric acids inhibit oligomerization of amyloid peptide 1-40 [51]. In addition, assessing the effect of microbial metabolites on aggregation of β-amyloid peptide 1-42, it was found that only valeric acid completely inhibited the formation of β-amyloid peptide oligomers [51]. But examination of the conversion of β-amyloid peptides into β-amyloid peptide fibrils revealed that both valeric and butyric acids inhibited the conversion of β-amyloid peptide 1-40 monomer to filamentous β-amyloid peptide [51]. These dose-dependent effects of intestinal metabolites show that increasing the amount of beneficial metabolite secreted by anti-inflammatory bacteria in the intestinal flora can promote the removal of β-amyloid peptide in the brain. In addition, in Alzheimer’s disease mouse model, acetate (a metabolite of *Bifidobacterium breve* strain A1) has been documented to ameliorate cognitive disturbances [75]. Interestingly, an improvement in behavioral deficits was also observed after oral administration of sonicated *Bifidobacterium breve* strain A1 homogenate [75].

## Probiotics and prebiotics in Alzheimer’s disease

Probiotics are bacteria that have a beneficial effect on the health of the recipient, while prebiotics are mainly fiber substances that serve as food for these bacteria [76]. Acute and chronic neuroinflammation is one of the key elements in amyloid accumulation and progression of Alzheimer’s disease [77, 78]. In this situation, pro/prebiotics, such as lactic acid bacteria and *Bifidobacterium*, have attracted attention as tools for suppressing neuroinflammation. However, data on the therapeutic effects of probiotics and prebiotics in Alzheimer’s disease are not extensive at present. Probiotic treatment with SLAB 51 cocktail in the mouse transgenic model of Alzheimer’s disease caused changes in the microflora, resulting in the altered content of metabolites of intestinaa such as short chain fatty acids that improved cognitive functions [79]. Other study supports the view that intestinal microflora can help prevent the development of Alzheimer’s disease, partly by supporting the production of short chain fatty acids that interfere with the formation of toxic soluble amyloid aggregates [51]. Oral administration of *Bifidobacterium breve* A1 ameliorated the cognitive decline observed in Alzheimer’s disease mice [75]. Gene profiling analysis revealed that the consumption of *Bifidobacterium breve* A1 suppressed the inflammation in the hippocampus and immune-reactive genes that are induced by amyloid [75]. A study conducted by the same group showed that *Bifidobacterium breve* A1 supplementation can have a beneficial effect on the cognitive function of older people with memory problems [80].

After 21 days of ingestion of a probiotic milk drink or placebo by 124 healthy adult volunteers, the cognitive function, as evaluated in two measures of memory was slightly worse in the probiotic group [81]. Another study showed that ingesting bioactive peptides in dairy products improves cognitive function [82]. Tryptophan-related dipeptides and new lacto peptides in fermented dairy products inhibit microglia activation and improve memory function and cognition [83, 84]. In addition, epidemiological studies involving 1056 people have revealed that consumption of cheese in the diet is associated with a lower prevalence of cognitive impairment [85]. Also, a study conducted on 1006 Japanese without dementia, aged 60-80 with 15 years of observation, showed that high consumption of milk and dairy products reduced the risk of dementia [86].

The data clearly indicate that healthy eating patterns characterized by high intake of prebiotics and probiotics in combination with other nutrients delay cognitive decline and reduce the risk of Alzheimer’s disease [87]. In addition, it has been shown that consumption of fermented milk product with probiotic not only affects normal brain activity [88], but also causes significant cognitive improvement in patients with Alzheimer’s disease [89]. These effects can be caused by the restoration of intestinal microflora, but also by the opposite effect to other pathological events associated with Alzheimer’s disease, such as oxidative stress [89, 90]. Recently, transgenic Alzheimer’s disease mice treated with probiotics have been shown to have better cognitive performance and reduced number of amyloid plaques in the hippocampus compared to untreated Alzheimer’s disease mice [91]. In another study, a similar effect on cognitive function in transgenic Alzheimer’s disease mice was documented after prebiotic administration [92]. Finally, the administration of a probiotic to rats was found to reverse the physiological and psychological abnormalities caused by the antibiotic ampicillin [93]. At this point, we can seriously consider modifying the intestinal microflora with pro-, pre- or antibiotics to obtain beneficial effects in the prevention and treatment of Alzheimer’s disease [94].

## CONCLUSIONS

The lack of causal treatment for Alzheimer’s disease is mainly due to the unknown disease etiology. Currently, there are several hypotheses regarding the etiology of Alzheimer’s disease, e.g. amyloid, ischemic [95] or hygiene [94] theory, which attempt to explain the mechanism of development of Alzheimer’s disease, but none of them now ultimately, solves the problem related to the etiology of the disease. Currently, the causative agents of this disease are based on many universally known mechanisms of neurodegeneration, including dysregulation of calcium homeostasis, abnormal accumulation of amyloid and dysfunctional tau protein, imbalance of neurotransmitters, necrotic and apoptotic neuronal death, disappearance of synapses, and neuroinflammation with pathological microglia and astrocyte activation in the brain, white matter changes and finally brain atrophy. The neuropathology of Alzheimer’s disease has long been considered an isolated brain disease with no relationship to other parts or organs of the body, but this view has now begun to change based on new data. Meanwhile, new scientific observations emphasize the important role of intestinal microflora in the normal functioning of the brain-gut-microbiota axis. These emerging studies represent the view that the gut microbiome probably affects brain development and functions, behavior and immunity in health and disease. Based on a few experimental and clinical studies, changes in the composition of the gut microbiome in neurodegenerative diseases, including Alzheimer’s disease, were also presented. Dysbiosis of the gut microbiome, often accompanied by fungi, jointly produces and releases e.g. neurotransmitters and pro-inflammatory mediators. The above molecules presumably increase the permeability of the intestinal mucosa and the blood-brain barrier, and this significantly intensifies neuroinflammatory reaction and amyloid generation and deposition in the brain. The above abnormalities associated with dysbiosis allow the entry of a large amount of bacterial amyloids, lipopolysaccharide and other molecules into the peripheral circulatory system and from the peripheral circulation to the brain. It is more likely that dysbiosis associated with toxic molecules can cause or support neurodegenerative processes, through disorders of the immune system, which is associated with excessive synthesis and accumulation of amyloid, deposition of dysfunctional tau protein and induction of chronic neuroinflammation in the brain tissue. This confirms the observations associated with neuropathological changes in the brain of Alzheimer’s disease patients, which begin 10-20 years before the appearance of clinical symptoms of the disease [1].

The two-way exchange of information between the intestinal microbiome and the brain suggests that intestinal contents may affect brain development, maturation, cognitive activity, functions and health. We should emphasize with a high probability that bacteria and fungi from the intestines can cause neuroinflammation and autoimmune reactions during the aging and Alzheimer’s disease development. A significant decline in cognitive function was confirmed in microbial transplanted mice from patients with Alzheimer’s disease relative to age. Regression analysis showed a relationship between cognitive decline and age of microflora transplanted mice from sick individuals. This directly proves that microflora transplanted mice from diseased patients had reduced cognitive function as recipients. Therefore, it has been suggested that the gut microflora affects the behavior of the host through its own metabolites. There is no doubt that in Alzheimer’s disease patients attempts to restore the gut microbiome to the boost composition in healthy adults can significantly slow the progression of neurodegeneration by reducing amyloidogenesis and/or neuroinflammation. Further research is needed to clarify whether bacterial-derived amyloids are involved in the triggering and/or progression of Alzheimer’s disease. However, more solid experimental evidence is still required to show that changes in the intestinal microflora are responsible for behavioral abnormalities. It is necessary to demonstrate the effect of microflora metabolites like amyloids on β-amyloid peptide generation and accumulation, tau protein dysfunction, neuroinflammation, neuronal death and vascular degeneration in various animal models so that the crosstalk between gut microbiome and its metabolites and neurodegeneration in Alzheimer’s disease can be fully understood. With the fast development of research in this field, the future boom of research for the treatment of Alzheimer’s disease can successfully focus on research on gut microbiome.
